# Commentary: Severe Sequelae to Mold-Related Illness As Demonstrated in Two Finnish Cohorts

**DOI:** 10.3389/fimmu.2017.01694

**Published:** 2017-12-07

**Authors:** Hauke Sebastian Heinzow, Birger Gustav John Heinzow

**Affiliations:** ^1^Department of Medicine B, University Hospital Muenster, Muenster, Germany; ^2^Department of Environmental Health Protection, State Office for Social Services, Kiel, Germany

**Keywords:** mould-related diseases, exposure, allergic reactions, mold, health effects

When reading the article by Tuuminen and Rinne, including two case reports on serious health sequelae due to mold exposure in two Finnish cohorts, we find serious flaws in the application of statistical and epidemiological methods, as well as the respective causal interpretations.

Observations of mucosal irritation, respiratory, neurological, skin, and other symptoms, in the first case report, which portrays a family, add to the current evidence, that dampness and mold in the indoor environment are a serious health risk, emphasizing the need for exposure cessation and prompt remediation (1). Unnecessarily and unfortunately for the family it took 4 years to find this most obvious cause.

Observations in the family case report are in accordance to international accepted consensus (1) that asthma is clearly associated with mold and dampness, (2) that for other illnesses there is sufficient evidence for an association, and (3) that the mechanisms causing dampness-related illness are still unknown (2).

The second case report relates to a school cohort that consists of 30 teachers and 50 students from a “mold-infested” school building with greatly elevated prevalence of autoimmune conditions and malignancies. The authors claim elevated incidence, e.g., 47.5-fold for lymphoma and 6-fold for breast cancer.

The cohort is very small, and the different diseases are compiled without ICD classification. Since exposure to mold is complex and variable and the causal agents are unknown, it is clearly impossible to define mold illness by a single ICD 10 classification. However, for many ICD 10 illnesses, the evidence for an association with mold and dampness has been continuously updated (2–6). Accordingly, a broad range of health effects has been attributed to mold and dampness in indoor environments like systemic infections, allergic and hypersensitivity reactions, and irritant or toxic reactions contributing to respiratory, immune, and neurological effects (7). For others like cancer, rheumatic and autoimmune diseases evidence is inadequate, insufficient, or simply lacking to support an association. Therefore, epidemiological (cross-sectional) studies are important instruments to advance our knowledge and to resolve existing controversies.

However, the study of the school cohort has many flaws and shortcomings that the conclusions must be disapproved. Fundamental requirements concerning data quality and statistical analysis are missing. Nominator and denominator for the comparison of incidence of cases are ill defined. None of the diseases is presented with the proper ICD 10 classification, e.g., lymphoma (ICD10: C81–C85) includes five and hypothyroid diseases and thyroiditis even more entries and subentries, all with different pathologies. The authors’ citation (8) for the corresponding incidence of the region is inappropriate and refers to a paper on chemical intolerance. Selecting a 20-year incidence period for teachers and 6 years for students without age and gender adjustment introduces a bias exaggerating the incidence for lymphoma. It is well recognized that a large random component may predominate disease rates across small cohorts and therefore comparison of incidence rates must include the confidence interval (95% CI) and all of the cases must involve the same type of cancer, or types of cancer scientifically proven to have the same cause (8). This is not the case and thus the claim for evidence of a cluster is not justified.

Another serious concern is related to the claimed evidence that toxic molds can be responsible for serious morbidity, even mortality. The paucity of data concerning the description of the indoor air quality and the humidity/mold problem and lack of exposure assessment adds to the limitations of the manuscript. Evidence on “toxic mold” exposure is lacking, at least the highly predictive semi-quantitative index of exposure to dampness and mold, based solely on visual and olfactory observation (9), should have been presented. No information on visible mold, elevated cfu/m^3^ in indoor air, odor, or indices of dampness, or elevated humidity is given. The listing of several different fungi in the mineral wool insulation material above a dubious cutoff value (cfu/g) without microscopy of mycelia and sporangia in the material is neither proof for mycotoxin production nor exposure. The hypothesis that the sequelae is due to mycotoxins is highly speculative. Mycotoxin production depends on water activity (aw above 0.95) and nutrient source (10). Furthermore, the flagships of mycotoxin producing molds in damp indoor environments *Aspergillus versicolor* and *Stachybotrys chartarum* were not found. Mineral wool insulation is a poor substrate, the formation of mycotoxins is possible but unproven. Since mycotoxins are not volatile but bound to spores and hyphen fragments and propagules they require a certain air velocity (m/s) for aerosolization. Significant exposure by liberation from mineral wool insulation behind a wall structure is questionable. Surprisingly, main symptoms of students are reported as flu-like symptoms and fatigue, whereas one would expect primarily respiratory and allergic symptoms and irritant/toxic mucosal reactions known to be associated with mold and dampness problem schools (11, 12).

In the discussion section, a highly speculative cause for the elevated incidence of thyroid disease is based on a single *in vitro* study with the bacterial ionophore amylosin (13). Maybe debatable for a cluster of eosinophilia–myalgia syndrome, this hypothesis has no foundation in this context. Moreover, other plausible environmental causes of thyroid disease (14) like iodine deficiency are completely ignored.

In addition, the alleged denial of mold-related illness in Finland ignores the existing literature. It is well accepted in Finland that indoor air problems are in many cases related to moisture and mold damage in structures and can cause diverse health symptoms (15). Nordic countries and especially Finland have a long tradition in public health research on mold and dampness (16–18). Standardized moisture performance assessments were already established in the 1990s and widely used (19).

The study addresses an important and interesting topic but is flawed because of poor and selective reporting. Based on case report data, the authors draw very strong conclusions without addressing limitations of the findings and considering alternative explanations. We interpret the oncological results as chance findings and consider the authors’ interpretation that mycotoxins are the cause of the other observed health effects highly speculative.

## Author Contributions

HH and BH contributed equally: analysis and interpretation of data, drafting of the manuscript, statistical analysis, and literature research.

## Conflict of Interest Statement

The authors declare the absence of any commercial or financial relationships that could be construed as a potential conflict of interest.
